# Reliability of three landmarking methods for dual inclinometry measurements of lumbar flexion and extension

**DOI:** 10.1186/s12891-015-0578-2

**Published:** 2015-05-20

**Authors:** Joy C MacDermid, Vanitha Arumugam, Joshua I Vincent, Kimberly L Payne, Aubrey K So

**Affiliations:** 1 Faculty of Health Sciences, University of Western Ontario, London, Ontario Canada; 2 Department of Surgery, University of Western Ontario, ON N6A 4L6 London, Ontario Canada; 3 Roth – MacFarlane Hand and Upper Limb Center, St. Joseph’s Healthcare London, London, Ontario Canada; 4 School of Rehabilitation Science, McMaster University, Hamilton, Ontario Canada

**Keywords:** Computerized dual inclinometer, Landmarking, Reliability

## Abstract

**Background:**

To examine the intra and inter-rater reliability of lumbar flexion and extension measurements attained using three landmarking methods for dual inclinometry.

**Methods:**

This was a repeated measures reliability study. Convenience sampling was used to obtain forty volunteer subjects. Two assessors measured a series of lumbar flexion and extension movements using the J-Tech™ dual inclinometer. Three different landmarking methods were used: 1) straight palpation of PSIS and L1, 2) palpation of PSIS and the site of the nearest 5 cm interval point closest to L1 and 3) location of PSIS and 15 cm cephalad. Upon landmarking, adhesive tape was used to mark landmarks and the inclinometer was placed on sites for three trials of flexion and extension. Tape was removed and landmarks were relocated by the same assessor (intra-rater) for an additional three trials; and this process was repeated by a second assessor (inter-rater). Reliability was determined using intra-class correlation coefficients.

**Results:**

Reliability within a set of three repetitions was very high (ICCs > 0.90); intra-rater reliability after relocating landmarks was high (ICCs > 0.80); reliability between therapists was moderate to high (0.60 > ICCs < 0.76). Assessment of flexion and extension movements by straight palpation of bony landmarks as in the Straight palpation of PSIS and L1 method (ICC: Flexion 0.60; Extension 0.74) was found to be marginally less reliable than the other two landmarking measurement strategies (ICC: Flexion 0.66; Extension 0.76).

**Conclusion:**

All three methods of land marking are reliable. We recommend the use of the PSIS to 15 cm cephalad method as used in the modified-modified Schobers test as it is the simplest to perform and aligns with current clinical practice.

## Background

Low back pain (LBP) is one of the most common musculoskeletal problems across the globe with an estimated lifetime incidence of 60-80 % [1]. The high costs of low back pain are borne by the government, insurance companies, and the general public in the form of medical treatments and impairment compensations [2, 3]. Range of motion (ROM) amongst other variables has been identified to be an indicator of impairment due to back pain [4].

A great deal of research has gone into examining different tools for measuring spinal mobility [5–8]. Tools available for clinical use include: plumb line, [9] goniometry, [10] fingertip to floor, [11] flexicurve, [12] tape measures, [13] visual-photographs, [14] and dual inclinometry. [15] At present, there is no conclusive evidence to advocate one method over another [16]. Radiographs are considered the gold standard for measuring absolute joint motion. [4, 7] However, performing a radiograph on every patient with LBP is costly and time-consuming in a clinical setting. Furthermore, range of motion measurements often reflect physical impairment or functional mobility in which case external measures of the ROM are more directly applicable.

One of the methods that has been used to measure joint mobility in research and clinical practice is computerized dual inclinometry (CDI). Previous studies have addressed the validity and reliability of the CDI [3, 7, 8, 17–19]. An examination by Nitschke et al. [3] showed dual inclinometry had acceptable intra-rater reliability results (flexion ICC = 0.90, extension ICC = 0.71), but fair to poor inter-rater values (flexion ICC = 0.67, extension ICC = 0.35). In a systematic review on the validity of instruments used to measure lumbar ROM, three out of the four studies included were on dual inclinometry [16]. The authors of that review concluded that there was little evidence to support current measures of lumbar ROM. The poor results were mostly attributed to differences between the raters, reflected by the accuracy and consistency of manual palpation of bony landmarks, and the handling of the inclinometer heads along the subject’s spine [20]. Nonetheless, dual inclinometry is considered to be more valid tool than modified-modified Schobers (MMS) [21] because the measurement results are expressed in degrees, which correlate with the angular motion of lumbar flexion and extension [22].

According to guidelines set out by the American Medical Association (AMA) in its 4th and 5th edition, compensation entitlements for patients with low back pain were based in part, on the impairment of back movement [23, 24]. It recommended the use of multiple measures including the CDI and the MMS. In the 6th edition however, ROM was removed from the list because of variability in results and lack of strong evidence supporting the validity and reliability of the ROM measures that are currently used [25]. When making judgments on a patient’s level of impairment, particularly when his/her future financial compensations hang in the balance, reliable methods are essential for determining impairment in spinal motion.

When making routine clinical decisions, a variety of measurement characteristics are important to consider. Ideally tools and the associated procedures to use them are reliable, valid, cost-effective & easy-to-use [7]. While a number of factors including rater skill, equipment limitations, patient selection and pain status may affect reliability of results, certainly landmarking is one of the major issues that affects the reliability and validity of joint motion measurements. Findings from previous reliability studies of the lumbar ROM measures suggest that palpation inaccuracies were the main source of error [3, 7, 8, 18, 19, 26, 27]. On the other hand, studies on palpation skills of clinicians in palpating and identifying spinal levels indicate great variability in terms of reproducibility and repeatability between clinicians [28–32]. Hence, one strategy for improving reliability is to study the impact of different landmarking techniques. The utility of a landmarking method will be based on producing accurate measurements with highest reliability with the least difficulty, time and cost. The purpose of this study is to examine the intra-rater and inter-rater reliability of lumbar flexion and extension measurements with a J-Tech dual inclinometer,^1^ using three different landmarking methods.

## Methods

### Study design

Repeated measures reliability study.

#### Subjects

Forty subjects (26 male, 14 female), ranging in age from 19 to 71 (mean = 34.2, SD = 14.5) were recruited by convenience sampling to participate in the study. (see Table 1) They were recruited from the community by posters and word of mouth. Any one above the age of 18 was selected. LBP was not a factor in the inclusion criteria for subject selection. They were excluded if they had experienced any trauma or surgery to their back or if they have any difficulties following instruction. The purpose of the study was explained to each subject and written informed consent was obtained before testing began. Subjects were also informed that participation in the study was completely voluntary, and they could withdraw at any time. This study was approved by the Health Sciences Research Ethics Board (HSREB) of the University of Western Ontario in London, Ontario, Canada.

**Table 1:** Subject demographics

Characteristics	Values
Subjects (n)	40
Male : Female	26:14
Mean Age in years (Mean and SD)	34.2(14.5)
Age range in years	19-71
Mean age in years (Male : Female)	33.7 : 34.6
Presence of low back pain (Yes : No)	23 : 17
Presence of low back pain (Male : Female)	14 : 9

### Landmarking techniques

The three landmarking techniques that were used. Landmarks were chosen based on our knowledge of anatomy of the lumbar spine and biomechanical measurement strategies used in the MMS [21].

#### Method 1- Straight palpation of PSIS and L1

To determine the lower landmark, subject’s PSISs were palpated and a line connecting both PSIS represents the level of S2. The upper landmark was determined by counting up spinous processes from S2 to L1.

#### Method 2 – PSIS to cephalad 10 or 15 cm

To determine lower landmark, the subject’s PSISs were palpated and a line connecting both PSIS represents the level of S2. The upper landmark was determined by counting up spinous processes from S2 to L1. The distance between S2 and L1 was measured in centimeters and rounded to either 10 or 15 cm based on proximity. An adhesive mark was then placed at the 10 or 15 cm level above the S2 level located.

### Method 3 – PSIS to 15 cm cephalad

To determine lower landmark, the subject’s PSISs were palpated and a line connecting both PSIS represents the level of S2. The upper landmark was determined by measuring exactly 15 cm above the located S2 level.

### Procedure

Each subject was asked to complete a set of three lumbar flexion and extension movements prior to testing as a warm-up procedure. Three different land marking methods (see below) were used to identify the start & end of the lumbar spine to be measured. The upper and lower spinal landmarks were marked by a horizontal line on a piece of adhesive tape.^2^ Adhesive marks were removed and re-landmarked for each set of data. The two heads of the dual inclinometer were placed at the low marked levels along the spine; the MASTER head at the upper landmark and the SLAVE head placed at the lower landmark. During each set, subjects performed a series of three alternating flexion and extension movements. All subjects were instructed to remove their shoes and to stand upright with feet shoulder-width apart, and both knees straight throughout the process. Subjects’ shirts were lifted up and clipped, in order to expose the lumbar spine. The instructions given to each subject were, “bend forward towards your toes starting with tucking your chin to your chest and slowly leaning down towards the floor” and “bend backwards as far as you can, with your hands on your waist and knees straight.” The J-Tech™ equipment was calibrated before each set of repeated movements. Two sets of data were obtained for lumbar flexion and extension for each rater and each landmarking method for a total of 160 sets. One set constituted three lumbar flexion movements and three lumbar extension movements that were alternating in nature. The adhesive marks were removed for each set and the order of rater was randomized.

### Statistical analysis

#### Relative reliability

Inter and intra rater reliability was calculated using intra class correlations (ICC) [33, 34]. An ICC value can range between 0 and 1 with zero indicating no reliability and 1 indicating perfect reliability.

## Results

### Intra-rater reliability

All three methods exhibited high to very high intra-rater reliability (ICC 0.78 to 0.93) for both lumbar flexion and extension values. (see Table 2) Extension measurements demonstrated high intra-rater reliability than flexion measurements. None of the three methods was superior to the other.

**Table 2:** ICCs for intra-rater reliability (Between sets)

Land marking technique	Raters	Flexion	Extension
***Method 1-*** *PSIS to L1*	A	0.80	0.89
	B	0.81	0.90
***Method 2*** *– PSIS to cephalad 10 or 15 cm*	A	0.90	0.93
	B	0.85	0.84
***Method 3*** *– PSIS to 15 cm cephalad*	A	0.88	0.94
	B	0.78	0.89

**Table 3:** ICCs for inter-rater reliability

Land marking technique	Flexion	Extension
*Method 1-* ***PSIS to L1***	0.60	0.74
*Method 2 –* ***PSIS to cephalad 10 or 15 cm***	0.66	0.76
*Method 3 –* ***PSIS to 15 cm cephalad***	0.66	0.76

### Inter-rater reliability

All three methods demonstrated high inter-rater reliability (ICC > 0.74) for extension measurements, while only moderate inter-rater reliability (ICC > 0.60) was observed for the three methods of landmarking for flexion measurements (see Table 3).

We used the following benchmarks in interpreting our results: 1 indicates perfect reliability, 0.90 to 0.99 = very high reliability; 0.70 to 0.89 = high reliability; 0.50 to 0.69 = moderate reliability; 0.26 to 0.49 = low reliability and 0.00 to 0.25 = little, if any, reliability [35].

## Discussion

The results of the current study indicate that regardless of the landmarking method utilized (methods 1, 2 or 3), dual inclinometry had good intra-rater reliability and fair to good inter-rater reliability for lumbar flexion and extension measurements. This adds on to the credibility of using dual inclinometer because reliable objective measures are fundamental to making data-driven clinical decisions. This study demonstrates reliable methods of measuring lumbar ROM are available. Since, lack of such evidence resulted in ROM being excluded from the measures that are required to calculate impairment due to back pain [25]. Since previous studies gave mixed messages on reliability of these measures, [4, 8, 18, 26] we do not suggest these findings are sufficient to change that decision. This study proposed a method to improve reliability of spinal motion measurements and there is value in adopting consistent methods to allow for greater comparability.

Currently, there is no clear evidence about the relevance of lumbar ROM in low back pain. This is commonly seen in routine clinical practice. Symptomatic patients might not show impaired range of motion while symptomatic patients might present substantial impairment of lumbar movements. The following is a classic example of this observation. Quack and colleagues (2007) compared MRI findings to ROM tests of the lumbar spine and found no clear relationship between the changes observed on a MRI to the ROM tests of the lumbar spine [36]. This is due to our very limited understanding of the relationship between impairment and function low back pain. Hence, studies on the relationship of spinal motion to function are needed to determine if this impairment should be considered important when evaluating impairment.

Nitschke et al. [3] demonstrated poor inter and intra-rater reliability for measurements of thoracolumbar flexion, extension, lateral flexion and rotation using a J-Tech CDI with respect to reliability. On the contrary, the current study has shown that with appropriate land marking techniques the reliability was fair to good. The differences in reliability reported across studies could be due to positioning. Investigators in the Nitschke et al. study used directions provided in the AMA guide (2nd edition) which does not clearly define the location for the master unit; it defines it as “mid-sacrum” which could bring about substantial variation in identifying this reference point between assessors. On the other hand in the current study we used an easy-to- identify landmark, PSIS which even a novice assessor can identify easily.

An important and common source of error while measuring lumbar range of motion could be to do with the preparation of subjects. Two important aspects of subject preparation are: exposing the lower spine to improve landmarking and considering the flexibility of hamstrings which could reduce the movement in pelvis and consequently in the lumbar spine [37]. In this study we exposed the lumbar spine by lifting and clipping the clothing. To improve flexibility of the hamstrings we made our subjects perform some practice repetitions which can increase the flexibility of the hamstrings considerably through ‘warm-up’ [38]. It is also believed that hamstring stretching could have a considerable effect on the lumbar spine because of its pelvic attachment; this might increase pain in patients with LBP and limit the actual lumbar ROM during the actual test. To this end, we did not incorporate a complete hamstring stretching protocol which may have affected spinal motion and also we ensured that the stretches were performed within the limits of pain.

The technique of applying the dual inclinometer also has potential sources of error. Positioning of the MASTER and SLAVE heads of the dual inclinometer may be problematic, including the ability to maintain a constant pressure with the heads against the skin. Measuring lumbar extension can be particularly awkward due to the tendency for the heaping up and folding of skin. Also, with highly flexible subjects, it can be difficult to position the heads to prevent them from colliding. Improvement can be made by moving either one of the measurements heads slightly lateral to prevent collision. This would still provide an accurate measurement without the perfect alignment of the heads because the unit measures the angle between the intersecting planes created at the heads.

Despite the mentioned sources of error, this study has shown dual inclinometry to be a reliable tool for measuring lumbar flexion and extension. Although validity was not tested, dual inclinometry provides its measurements in degrees, which better represents the true spinal angular movement than the linear skin distraction method of MMS. Moreover the criterion validity of dual inclinometer has been tested against the gold standard of radiographic measurements in previous studies [39, 40]. However, additional research is needed to confirm the validity of the dual inclinometry techniques mentioned in this study for measuring lumbar spinal movement.

There are many different landmarking techniques currently used by clinicians to locate a specific spinal level. However, the reliability between therapists to find the same spinal level tends to be problematic [27]. Sources of error include the level of training of the therapist, how well the lumbar spine is exposed away from clothing, and the varying distances for individuals of the bony landmarks to the surface of the skin. However, studies have shown that even highly experienced clinicians have low reliability in accurate ascertainment of spinal level, suggesting that measurement strategies based on palpation have inherent limitations [32]. For this reason and knowing that, most patients in this study fell into the 15 cm distance that is currently used in the MMS. A measurement strategy that utilizes this method may produce more reliable results when one considers the wide spectrum of potential testers that might use inclinometry for spinal evaluation. A limitation of this method is that it does not represent the same portion of the spine for people of different heights. Whether this means the measurement is less valid or less related to function in different populations or across people of different heights, needs further study. A limitation of this current study is its inability to calculate values for absolute reliability which would have made the study even more clinically relevant. We recommend future studies to determine Standard Error of Measurement (SEM) values to determine absolute reliability.

## Conclusion

All three landmarking techniques have almost the same reliability. However, from a clinical standpoint, the PSIS to 15 cm cephalad method as used in the modified-modified Schobers test, is recommended, as it is the simplest to perform and aligns with routine clinical practice. Dual inclinometry can produce clinically reliable measurements provided a reliable landmarking strategy is employed.

## Footnotes

1 Tracker M.E. J-Tech Medical Industries, Utah, USA

2 3 M™ Micropore™ Medical Tape, 3 M in Canada, London ON.
